# Does decreased visual attention to faces underlie difficulties interpreting eye gaze cues in autism?

**DOI:** 10.1186/s13229-020-00361-2

**Published:** 2020-07-21

**Authors:** Jason W. Griffin, K. Suzanne Scherf

**Affiliations:** Department of Psychology, Pennsylvania State University, University Park, PA 16802 USA

**Keywords:** Eye tracking, Face processing, Joint attention, Gaze perception, Gaze following, Adolescent

## Abstract

**Background:**

Shifts in eye gaze communicate social information that allows people to respond to another’s behavior, interpret motivations driving behavior, and anticipate subsequent behavior. Understanding the social communicative nature of gaze shifts requires the ability to link eye movements and mental state information about objects in the world. Autism spectrum disorder (ASD) is characterized by atypical sensitivity to eye gaze cues, which impacts social communication and relationships. We evaluated whether reduced visual attention to faces explains this difficulty in ASD.

**Methods:**

We employed eye-tracking technology to measure visual attention to faces and gazed-at objects in a 4-alternative forced choice paradigm in adolescents with ASD and typically developing (TD) adolescents. Participants determined the target object that an actor was looking at in ecologically rich scenes. We controlled for group differences in task engagement and data quality.

**Results:**

In the Gaze Following task, adolescents with ASD were relatively impaired (Cohen’s *d* = 0.63) in the ability to identify the target object. In contrast to predictions, both groups exhibited comparable fixation durations to faces and target objects. Among both groups, individuals who looked longer at the target objects, but not faces, performed better in the task. Finally, among the ASD group, parent SSIS-Social Skills ratings were positively associated with performance on the Gaze Following task. In the Gaze Perception task, there was a similar pattern of results, which provides internal replication of the findings that visual attention to faces is not related to difficulty interpreting eye gaze cues. Together, these findings indicate that adolescents with ASD are capable of following gaze, but have difficulty linking gaze shifts with mental state information.

**Limitations:**

Additional work is necessary to determine whether these findings generalize to individuals across the full autism spectrum. New paradigms that manipulate component processes of eye gaze processing need to be tested to confirm these interpretations.

**Conclusions:**

Reduced visual attention to faces does not appear to contribute to atypical processing of eye gaze cues among adolescents with ASD. Instead, the difficulty for individuals with ASD is related to understanding the social communicative aspects of eye gaze information, which may not be extracted from visual cues alone.

## Background

Visual attention to faces is critical for social communication because the face is rich with cues signaling the intentions, emotions, and goals of others. Eyes in faces are especially revealing and the ability to process information about *eye gaze* is foundational to human social interactions (see [1]). Even from the first days of life, human infants possess a rudimentary ability to follow another person’s eye gaze [2]. Shifts in gaze provide information about objects and people in the world. Specifically, the ability to detect what or who another person is looking at provides information about the importance and relevance of things and people in the world. Shifts in gaze also provide more subtle cues about social interactions and communication like the visual perspective and/or social status of another person and whether they are trying to be deceptive (see [1]). Sensitivity to eye gaze allows people to respond to another’s behavior, make attributions about motivations driving the behavior, and anticipate subsequent behavior. Therefore, even relatively subtle impairments in sensitivity to eye gaze cues could have important ramifications for social communication and relationships.

### Autism and impaired eye gaze processing

Autism spectrum disorder (ASD) is a neurodevelopmental disability that is characterized by atypical sensitivity to eye gaze cues. Individuals with ASD often exhibit abnormal eye contact and have difficulty understanding and using eye gaze cues. Importantly, this is a diagnostic feature of the disability [3]. These deficits in sensitivity to eye gaze were originally studied empirically in episodes of joint attention (i.e., shared attention to objects) with other people (e.g., [4, 5]) and may contribute to difficulties that people with ASD have in developing and maintaining social relationships, adjusting their behavior to suit different social contexts, making friends, being interested in people, and in social-emotional reciprocity. Therefore, it is essential to understand the patterns of behavior and underlying mechanisms that contribute to the deficits in sensitivity to eyes and eye gaze cues.

More recently, researchers have investigated atypical sensitivity to eye gaze cues by measuring visual attention using eye-tracking while participants view photographs or movies of people. The central question has been to evaluate whether individuals with ASD exhibit *reduced* visual attention to faces, given the clinical observation that many show reduced eye contact in social interactions. The inference is that less time looking at faces provides fewer opportunities to learn about and interpret eye gaze and other non-verbal communicative behaviors from the face. In spite of the clear prediction, the research findings are quite mixed. Specifically, group differences between individuals with ASD and typically developing (TD) individuals in visual attention to faces exist, but the magnitude of these differences is inconsistent and largely dependent on experimental factors like the nature of the task, instructions and stimuli, and age of the participants (for review see [6, 7]). Also, most of the work employs passive viewing paradigms with no strategy for empirically evaluating the central assumptions, namely, that the duration of visual attention is a direct reflection of information processing about gaze (i.e., longer duration equals better and more processing of gaze) and of how eye gaze functions for social communication (i.e., understanding the communicative intent of gaze shifts).

There are a few studies that measure sensitivity to the *social communicative* aspects of eye gaze information. Riby and colleagues designed a paradigm in which participants must interpret the communicative intent of the eye gaze cues [8, 9]. In these tasks, participants view photographs of an actor looking at a single object in a complex scene. Participants are required to verbalize the name of the gazed-at object. In order to successfully identify and name the gazed-at object, participants must have *referential understanding* of the visual behavior of the actors in the scene. This requires an understanding that visual behavior is directed toward objects/content (i.e., it is not abstract in nature) and that it involves the mental experience of seeing something [10, 11]. In other words, it requires establishing a psychological connection between the looker and the content [12]. Having methods to assess referential understanding of gaze cues is critical because gaze following can reflect sensitivity to a predictive spatial cue (i.e., head or gaze direction indicates something interesting is about to happen over there; [13]) in the absence of comprehension about the psychological relation between the looker and target. Among TD individuals, referential understanding of eye gaze cues develops over the first 2 years of life, as infants learn that open eyes, not closed eyes or simple head direction, provide communicative information about the content (e.g., [12, 14]).

The studies investigating referential understanding of gaze cues in adolescents with ASD indicate that they are less skilled at generating labels to identify gazed-at objects than are TD adolescents, which suggests that there is difficulty perceiving and/or interpreting eye gaze cues [8, 9]. Adolescents with ASD also show reduced visual attention to both the face and the gazed-at objects (i.e., the referential locus of the gaze cues) [9]. Unfortunately, the researchers did not evaluate an association between visual attention and task performance, which would have helped assess the underlying assumption that shorter looking times to faces are related to worse performance.

### Current study

Given these limitations in the current literature, the goal of this study was to evaluate the notion that reduced visual attention to faces contributes to the difficulty understanding eye gaze cues in ASD. To address this goal, we measured visual attention to faces using eye tracking as adolescents with ASD and TD adolescents determined the communicative intent of eye gaze cues in two tasks. In both tasks, participants had to view ecologically rich scenes, determine the specific object that an actor was looking at, and pick out the label for the object in a 4-alternative forced choice (4AFC) task. We also measured visual attention to the target object, which reflects the referential intent of the gaze cue, as well as the accuracy with which participants identified this object in the 4AFC task. In the *Gaze Following Task*, we assessed whether adolescents with ASD could follow and interpret dynamic shifts in eye gaze. In the *Gaze Perception Task*, we assessed whether adolescents with ASD could compute, infer, and interpret eye gaze trajectory from photographs like those used in prior studies (e.g., [9]). Critically, by assessing visual attention to faces and target objects as well as behavioral performance in the task, we could measure the extent to which varying levels of attention are associated with relatively impaired abilities to use the eye gaze cues to solve a social communication problem.

Importantly, atypical visual attention to faces and eye gaze sensitivity is most frequently reported in infants and children with ASD; however, the developmental pattern of change in sensitivity to eye gaze cues in ASD is largely unclear [7]. We focused on evaluating sensitivity to eye gaze cues during *adolescence* in people with ASD for several reasons. First, adolescence is a vulnerable time for the development of the face processing system in autism (see [15]). Second, the demands for understanding and interpreting eye gaze cues in particular may be changing in adolescence when the very nature of social relationships changes.

We hypothesized that adolescents with ASD would show reduced ability to identify the target objects in the 4AFC tasks and reduced visual attention to faces. If reduced visual attention to faces is the underlying mechanism driving atypical processing of eye gaze cues, then less attention to faces should predict worse performance on the task, especially for the adolescents with ASD.

## Method

### Participants

A total of 89 participants were tested in this study. This included 40 adolescents with ASD and 49 TD adolescents. All individuals with ASD participated in a larger intervention study (NCT02968225 [16]). The data described here are part of the baseline assessment. Comparisons with TD adolescents have not been reported.

Participants were between the ages of 10 and 18 years and had a Full-Scale IQ between 70 and 130 (as assessed with the KBIT-2 [17]), normal vision, and hearing (with correction), were native English speakers, consented, and were compliant with the testing procedures. The full inclusion criteria and recruitment information are reported elsewhere [16]. TD participants were over-recruited and matched to the participants with ASD on age, sex, and Full-Scale IQ using propensity score matching [18]. The final sample for the analysis included a total of 70 adolescents (35 ASD, 35 TD). The demographic characteristics are in Table 1.

**Table 1:** Participant demographic characteristics

	ASD	TD	*p*
*N*	35	35	
Age	13.5 (2.7)	13.9 (2.1)	ns
Sex	29 M, 6 F	24 M, 11 F	ns
VIQ	96.8 (17.7)	108.1 (10.1)	< .01
PIQ	102.4 (14.2)	101.7 (13.6)	ns
FSIQ	100.1 (15.7)	106.5 (11.6)	ns
ADOS-2 Total	14.1 (4.4)	NA	
ADOS-2 SA	10.6 (3.8)	NA	
ADOS-2 RRB	3.49 (1.7)	NA	
ADOS-2 Total CSS	7.7 (1.8)	NA	
SSIS–Social Skills	79.0 (14.8)	NA	
SSIS–Problem Behavior	118.8 (13.5)	NA	
SRS-2 Total	75.8 (10.1)	NA	

*Note.* Cells represent mean (SD). *ADOS-2* Autism Diagnostic Observation Schedule (2nd Ed.), *SA* social communication, *RRB* repetitive and restricted behavior, *CSS* Calibrated Severity Score, *VIQ* verbal IQ, *PIQ* Performance IQ, *FSIQ* Full Scale IQ; *SSIS* Social Skills Improvement System (parent report), *SRS-2*Social Responsiveness Scale (2nd Ed)

### Stimuli

We created the stimuli for two tasks: the Gaze Following and Gaze Perception tasks. In both tasks, each stimulus depicted an unfamiliar adult directing gaze to a single target object in a complex scene. In addition to the target object, each stimulus also contained a plausible non-target object (i.e., near the target object but not gazed at) and several implausible objects (i.e., farther away from target object and not gazed at). The full details about the creation and validation of the stimuli are described elsewhere [19]. The stimuli are curated on Databrary and can be accessed for research purposes (10.17910/b7.884).

#### Gaze following videos

**Fig. 1:**
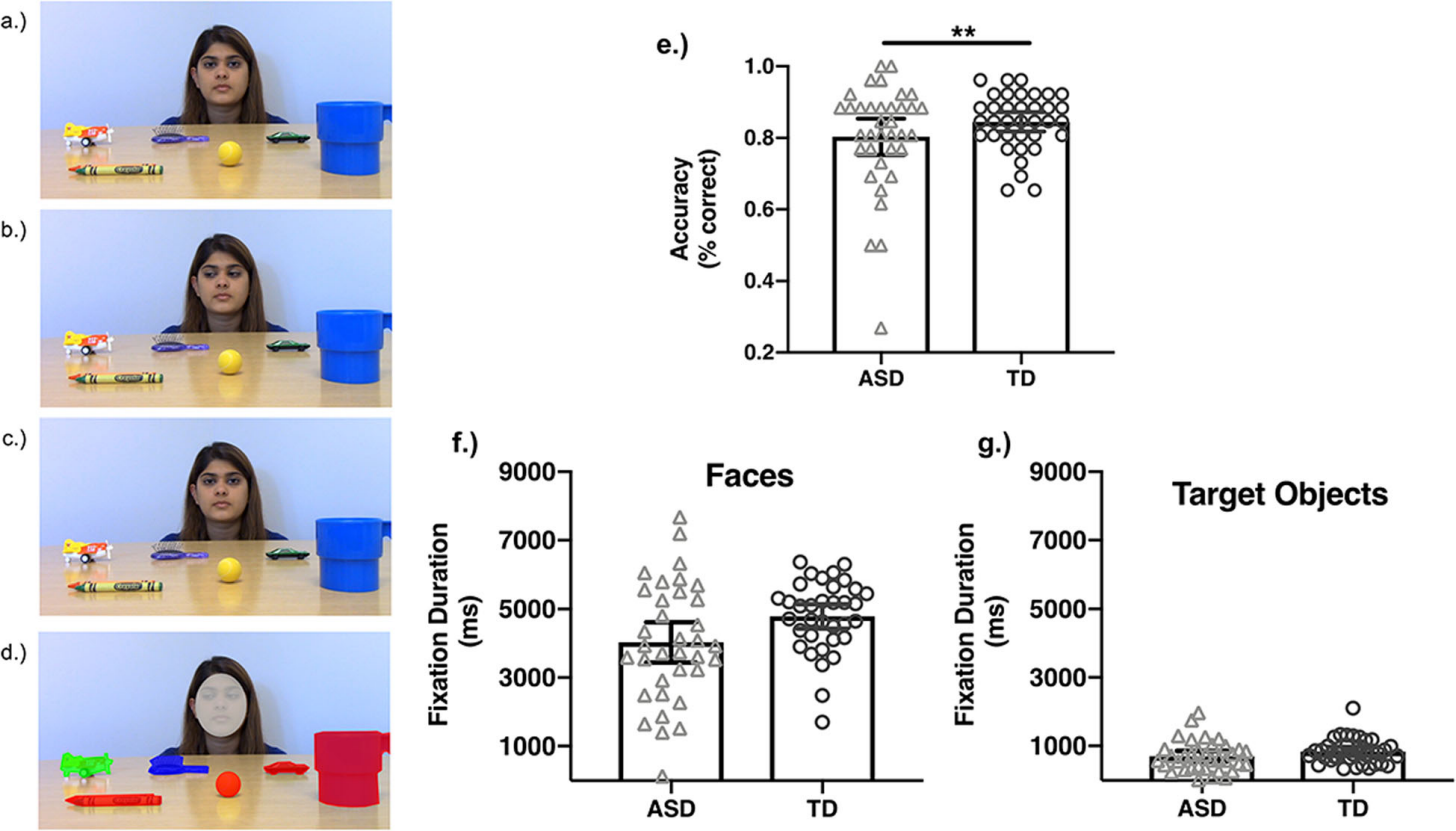
Gaze following stimuli and results. The left panel shows still frame images from one of the gaze following videos to illustrate the sequence of events. The event begins with the actor looking directly into the camera (**a**); she executes a gaze shift to the target object (**b**) and shifts gaze back toward the camera (**c**). The areas of interest for the fixation analyses are illustrated in **d**. The target object is in green, the plausible non-target object is in blue, and the implausible non-target objects are in red. The face is in grey. Task performance (% correct) is plotted as a function of group (**e**). Fixation durations to faces (**f**) and target objects (**g**) are plotted separated by group. Error bars reflect 95% confidence intervals. All analyses used mixed-effects modeling of trial-level data; however, for visualization these plots reflect the mean-level data. **p < .01

This task was designed to evaluate whether adolescents with ASD exhibit a relative impairment in the ability to follow shifts in eye gaze for the purpose of referential understanding. The stimuli were modeled after those used to evaluate sensitivity to gaze shifts and joint attention in infants and toddlers [20, 21]. In each video, a single female actor sat on a chair behind a table that occluded her body, which prevented social cueing from her body to indicate her gaze position. There are 6–8 small nameable objects (e.g., crayon, car) in each video. The position of the objects rotated across stimuli. Each video begins with the actor looking straight into the camera for 2 s as if making eye contact with the participant in an episode of joint attention (see Fig. 1a). Next, the actor shifts her gaze toward the target object (~ 500 ms) and holds her gaze on the target object for 4 s (see Fig. 1b). Finally, the actor shifts her gaze back toward the camera (~ 500 ms) and holds her gaze at the camera for 2 s (Fig. 1c). Each video lasts approximately 9 s. There were 26 videos.

#### Gaze perception images

This task was designed to evaluate whether adolescents with ASD are impaired in the ability to interpret eye gaze trajectory from photographs like those used in prior studies. These stimuli were inspired by those created by Riby et al. [9] and included digital color photographs of actors in complex naturalistic visual scenes (see Fig. 3a, b). All image sizes were standardized (1100 × 825 pixels). There were 40 images.

### Clinical assessments

All ASD diagnoses were confirmed via the Autism Diagnostic Observation Schedule, Second Edition (ADOS-2 [22]) and expert clinical evaluation. Because of the large age range of the participants, both modules 3 and 4 were used to assess the ASD diagnosis. As a result, we computed calibrated severity scores (CSS) for module 3 [22] and module 4 [23] for statistical analysis. Social skills and problematic behaviors were evaluated using both the self- and parent-report versions of the Social Skills Improvement System (SSIS [24]). On the parent-reported SSIS, higher scores reflect more behaviors. So higher SSIS-social scores indicate more social skills, whereas higher SSIS-problem behavior scores reflect more of these behaviors. In addition, parents of adolescents with ASD reported on autism-like behaviors in their child using the Social Responsiveness Scale, Second Edition (SRS-2 [25]). Higher SRS-2 total scores reflect more autism-like symptoms.

### Procedure

All parents and/or older adolescents provided written consent and younger adolescents provided written assent according to procedures approved by the Institutional Review Board (IRB) at Pennsylvania State University. Participants completed the IQ assessment prior to the eye gaze tasks. Participants were compensated $20/h.

Prior to the data collection session, all participants underwent an orientation procedure to familiarize them with the experimenter and eye-tracking lab, provide them with clear expectations about the sequence of the tasks, and help them understand how the eye tracking unit works to optimize data collection procedures (see Supplementary Materials).

TobiiPro Studio was used to display the stimuli and collect data. Fixation data were collected using a Tobii X2-60 eye tracker (sampling rate of 60 Hz, ~accuracy of 0.4°, precision of 0.34°) that was integrated with a Dell Optiplex 7040 computer and 24-in. monitor (60 Hz refresh rate). Participants were positioned ~ 65 cm from the display monitor. The tasks were administered in a fixed order beginning with a 9-point calibration procedure (see Supplementary Table 1). Any calibration point beyond the 1° confidence area was re-calibrated.

The Gaze Following stimuli were displayed for 9000 ms and the Gaze Perception for 4000 ms on each trial. Participants were instructed to view each item and identify the object that the actor was looking at. Note that these instructions emphasize the use of gaze information for the purpose of referential understanding (i.e., understanding that visual behavior involves the mental experience of seeing something and is directed toward objects/content).

Immediately after the stimulus was displayed, participants were presented with 4AFC answers on a separate screen. The choices included the target object, a plausible non-target object, and two implausible objects. The order of the items was counterbalanced across trials. Participants had an unlimited amount of time to select an answer using the mouse. Following the response, a 1000-ms fixation cross was displayed prior to the next trial. Participants completed three practice trials before each task. Participants received a 45-s break after every 7 trials and a 2-min break halfway through each task.

Parents of participants with ASD completed the clinical assessment surveys while the adolescents were being tested in the eye-tracking paradigm. Participants with ASD completed the self-report surveys after the eye-tracking protocol with the assistance of a member of the research team.

### Data analysis

#### 4AFC performance

Accuracy was the primary dependent variable.

#### Eye tracking data quality

To measure the ability of the eye tracker to accurately estimate the location of gaze, we measured the number of gaze samples in which the eye-tracker successfully estimated the gaze position on the display monitor (i.e., gaze capture). We computed total fixation duration to the stimulus (versus whole display) for each trial (i.e., stimulus fixation). We assessed group differences in these metrics and included them as trial-varying covariates in all statistical models when present.

#### Visual attention

We computed fixation duration (in milliseconds) for the entire stimulus and within each defined area of interest (AOIs). Raw gaze data were preprocessed using the Tobii identification velocity threshold filter with a minimum fixation duration of 100 ms. Gaze samples were only included when there was recordable information from at least one eye *on the stimulus*.

All AOIs were manually constructed for each stimulus with hard boundaries (see Figs. 1d and 3d) from the first frame of the video (see Fig. 1d). For the objects and faces in the Gaze, following videos, the AOI coordinate positions were extrapolated across the remaining frames for the rest of the video. The manual adjustment of the AOI coordinates across frames for the faces accommodated subtle head movements. For each participant, the duration of all fixations within an AOI was summed to generate a *total fixation duration* for each stimulus in each task.

#### Statistical analysis

We analyzed the data using *lme4* for the statistical software program R ([26]; R [27]). To maximize statistical power and account for non-independence (e.g., repeated measurement) in observations**,** we used linear mixed-effects modeling to evaluate group differences in task performance and visual attention for each task separately. Participant and stimulus item were included as crossed random effects and diagnostic group (ASD, TD) was the fixed factor (reference = TD). This approach is powerful because it eliminates the need to pre-average the data across trials, which can remove important variance; it adjusts estimates for repeated sampling; and it explicitly models variance [28]. We modeled each trial for each participant, which reduces the impact of extreme and missing observations, while not overfitting the data. As a result, we did not eliminate or alter any data points because of extreme status (i.e., outlier) or interpolate missing data. We evaluated potential group differences in the variance of each measure using the Levene’s test of homogeneity. We used a binomial distribution to fit the accuracy models and a gaussian distribution to fit the fixation duration models. All models included a random intercept to account for individual-level variability in outcome measures.

We also used linear mixed-effects modeling to evaluate whether fixation duration predicts accuracy (main effect) and whether this association is influenced by group (duration × group interaction). Significant interactions were interpreted by assessing simple main effects of fixation duration within each group separately.

Finally, we used a similar linear-mixed modeling approach to assess whether underlying social deficits and/or autism symptoms predict accuracy or fixation duration (total, faces, target objects) in the ASD group. We conducted separate analyses for each of the following measures: ADOS-2 Total CSS scores, SSIS-Social Skills, SSIS-Problem Behaviors, and SRS-2 Total scores.

## Results

### Participants

Prior to matching, one participant with ASD was removed for poor calibration. The groups did not differ in age *t*(68) = 1.14, *p* = .26, sex *χ*^2^(1) = 1.24, *p* = .26, or Full Scale IQ (FSIQ), *t*(68) = 1.93, *p* = .06; however, they did differ in verbal IQ (VIQ), *t*(68) = 3.28, *p* < .01 (see Table 1). Importantly, verbal IQ was not correlated with accuracy in either task (*p* > .05). In the Gaze Following task, VIQ was related to the duration of fixations to the target object (*p* < .05). As a result, we did a secondary analysis with VIQ as a covariate and confirmed that the pattern of results was unchanged. The correlation matrix evaluating associations among the clinical measures in the ASD group is provided in Supplementary Table 3.

### Gaze following

The group means for each dependent variable are reported in Table 2 and displayed in Fig. 1. Parameters from the statistical models are reported in Table 3.

**Table 2:** Accuracy and fixation data for both eye gaze tasks

	Gaze following		Gaze perception	
	ASD	TD	ASD	TD
Accuracy (%)	82% (14%)	89% (7%)	80% (12%)	88% (9%)
Fixation duration—faces	3932 (1755)	4691 (1065)	961 (591)	1122 (348)
Fixation duration—target objects	683 (444)	820 (366)	440 (230)	550 (203)

*Note.* Cells represent mean (SD). Accuracy is reported as percent correct. Fixation times reported as total fixation duration in milliseconds

**Table 3:** Parameter estimates for models evaluating group differences in each task

		*b/OR*	SE	*t*	Lower CI	Upper CI	*p*
Gaze following task							
*Model 1 (main effect on DVs)*							
Task performance (group)		0.50	0.13	− 2.71	0.31	0.83	**0.01**
Face fixation (group)		− 0.19	0.18	− 1.07	− 0.55	0.16	0.29
Target object fixation (group)		− 0.05	0.09	− 0.63	− 0.22	0.11	0.53
*Model 2 (fixation predicting accuracy)*							
Stimulus fixation		1.19	0.07	3.07	1.07	1.33	**0.002**
Stimulus fixation × group		1.02	0.09	0.16	0.84	1.22	0.87
*Model 3 (fixation predicting accuracy)*							
Face fixation		1.04	0.07	0.54	0.90	1.19	0.58
Face fixation × group		1.16	0.11	2.25	0.96	1.39	0.12
*Model 4 (fixation predicting accuracy)*							
Target object fixation		10.14	2.62	8.96	6.11	16.82	**< 0.001**
Target object fixation × group		2.12	1.04	1.53	0.81	5.56	0.13
Gaze perception task							
*Model 1 (main effect on DVs)*							
Task performance (group)		0.39	0.10	− 3.51	0.23	0.66	**< 0.001**
Face fixation (group)		− 0.05	0.08	− 0.61	− 0.21	0.11	0.55
Target object fixation (group)		− 0.06	0.04	− 1.35	− 0.15	0.03	0.18
*Model 2 (fixation predicting accuracy)*							
Stimulus fixation		1.04	0.11	0.33	0.84	1.28	0.74
Stimulus fixation × group		1.23	0.24	1.07	0.84	1.80	0.27
*Model 3 (fixation predicting accuracy)*							
Face fixation		0.92	0.10	− 0.71	0.74	1.15	0.48
Face fixation × group		1.49	0.27	2.2	1.04	2.14	**0.03**
TD main effect		0.77	.13	− 1.58	0.55	1.07	0.11
ASD main effect		1.02	.15	.17	0.77	1.36	0.87
*Model 4 (fixation predicting accuracy)*							
Target object fixation		1.95	0.32	4.06	1.41	2.69	**< .001**
Target object fixation × group		0.23	0.08	− 4.23	0.12	0.46	**< .001**
TD main effect		8.24	2.96	5.87	4.08	16.7	**< .001**
ASD main effect		1.1	0.21	0.48	0.76	1.59	0.63

*Note. TD* is reference group, *OR* odds ratio, *DV* dependent variable, *CI* 95% Confidence Interval

#### Accuracy

Figure 1e shows that adolescents with ASD exhibited impaired performance in the ability to follow eye gaze to identify gazed-at objects, which was evident in the significant main effect of group (see Table 3). The variance of task performance was comparable across groups, *F*(68,1) = 3.29, *p* = .07.

#### Eye tracking data quality

Our ability to capture eye gaze positions was comparable for both groups (*b* = − 0.05, se = 0.02, 95% CI [− .09, 0.00], *p* = .052).

#### Visual attention

Figure 1f, g shows the mean fixation duration to faces and target objects as a function of group.

#### Task engagement

Adolescents with ASD spent less time fixating the stimuli (*M* = 6110 ms, SD = 1981 ms) compared to TD adolescents (*M* = 6906 ms, SD = 988 ms) (*b* = − 0.80, se = 0.37, 95% CI [− 1.53, − 0.06], *p* = .03). The variance of these total fixation times was also different across groups, *F*(68,1) = 8.16, *p* = .005 (see Supplementary Figure 1). As a result, we included total fixation time for each stimulus as a covariate to control for this group difference in task engagement.

#### Faces

On average, adolescents with ASD and TD adolescents both looked at the face for approximately half the duration of the video (see Fig. 1f). In contrast to predictions, there was no main effect of group (see Table 3). This indicates that on average, adolescents with ASD and TD adolescents share similar amounts of social visual attention to faces. However, we did observe a significant difference in the variance of fixation durations to faces, *F*(68,1) = 6.11, *p* = .02 (see Supplementary Figure 1). There was larger variance among the adolescents with ASD, indicating greater individual differences in looking times to faces.

#### Target objects

Overall, fixations to target objects were much shorter than to faces for both groups (see Fig. 1g). There was no main effect of group on the duration of fixations to target objects (see Table 3), indicating similar allocation of amount of attention to target objects across groups. There were also no group differences in the variance of these fixation durations to target objects, *F*(68,1) = 0.91, *p* = .314.

#### Relating eye tracking to behavioral data

Total fixation duration to the stimuli significantly predicted task performance in both groups equally (see Table 3). Importantly, there was no group × fixation duration interaction, indicating that there were no differences in the magnitude of this association across groups.

**Fig. 2:**
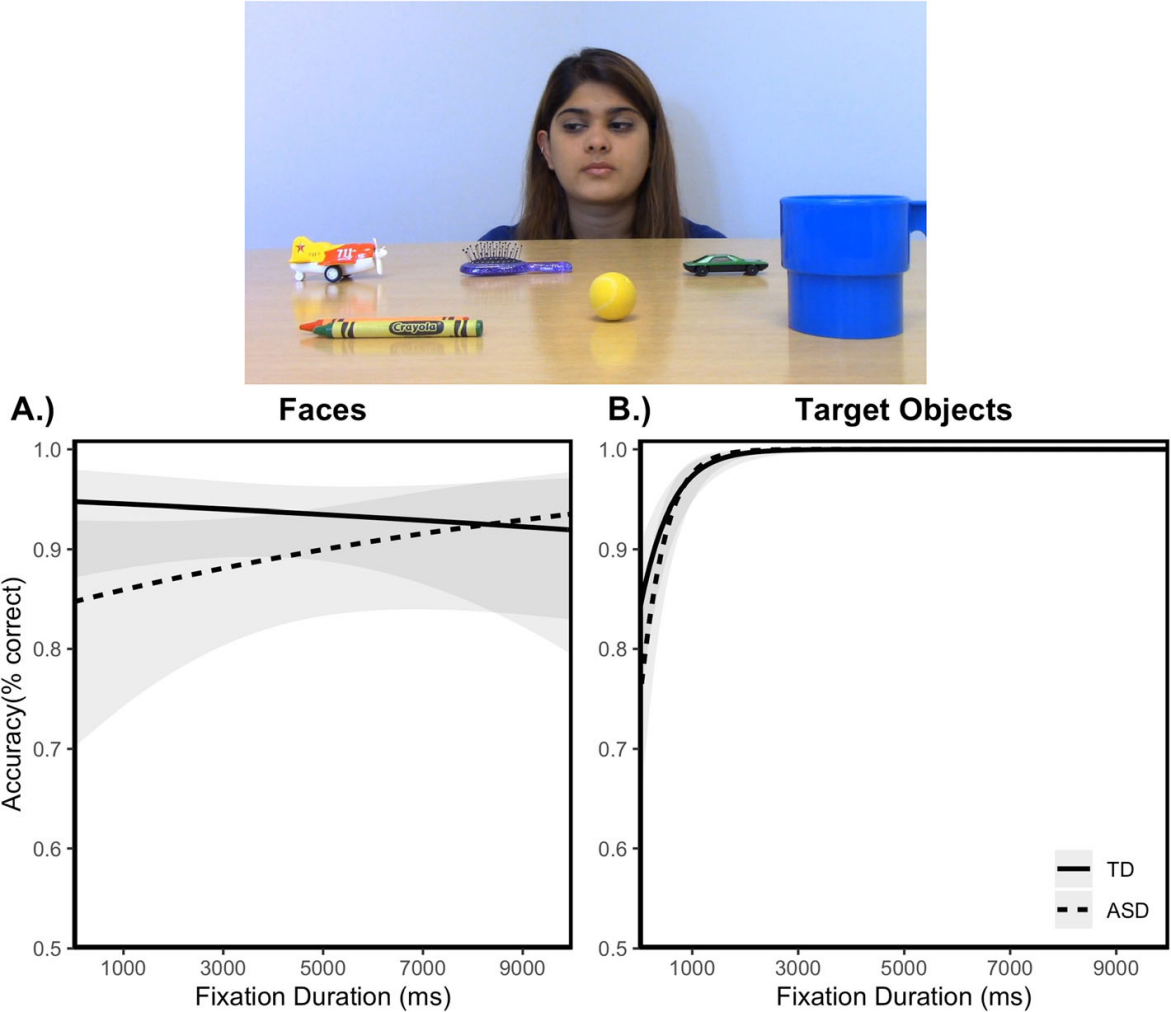
Association between visual attention and behavior during gaze following. Probability of identifying correct target object as a function of fixation duration to *face*, plotted as a function of group (**a**). There was no association between fixation duration to faces and performance in either group. Probability of identifying correct target object as a function of fixation duration to *target object*, plotted as a function of group (**b**). Longer fixation duration to target objects was associated with improved task performance for both groups. Shaded region reflects 95% confidence intervals. All plots reflect model-predicted relationships based on the mixed-effect models

Figure 2 shows the association between looking time to the face (a) and target object (b) AOIs and accuracy as a function of group in the Gaze Following task.

#### Faces

Neither group exhibited differential task performance as a function of fixation duration to faces (see Fig. 2a). There was no main effect of fixation duration or fixation duration × group interaction on task performance (see Table 3).

#### Target objects

In contrast, Fig. 2b shows that for both groups, longer fixation durations to gazed-at objects were associated with an increased likelihood of correctly identifying the target object. There was a main effect of fixation duration on task performance, but no fixation duration × group interaction (see Table 3).

### Relating clinical assessments to eye-tracking and behavior data

Social skills (parent reported SSIS) predicted task performance (*b* = 1.03, se = 0.01, 95% CI [1.01, 1.06], *p* < .01). Specifically, adolescents with more social skills looked longer at target objects. Also, ADOS-2 Total CSS scores negatively predicted fixation duration to target objects (*b* = − 0.10, se = 0.04, 95% CI [− 0.18, − 0.02], *p* = .02; see Supplementary Table 2). Adolescents with more severe ASD (i.e., higher CSS scores) exhibited shorter looking times to target objects. No other measures of autism symptoms or problematic behaviors predicted task performance or fixation duration to the stimuli, faces, or target objects.

### Gaze perception

#### Accuracy

**Fig. 3:**
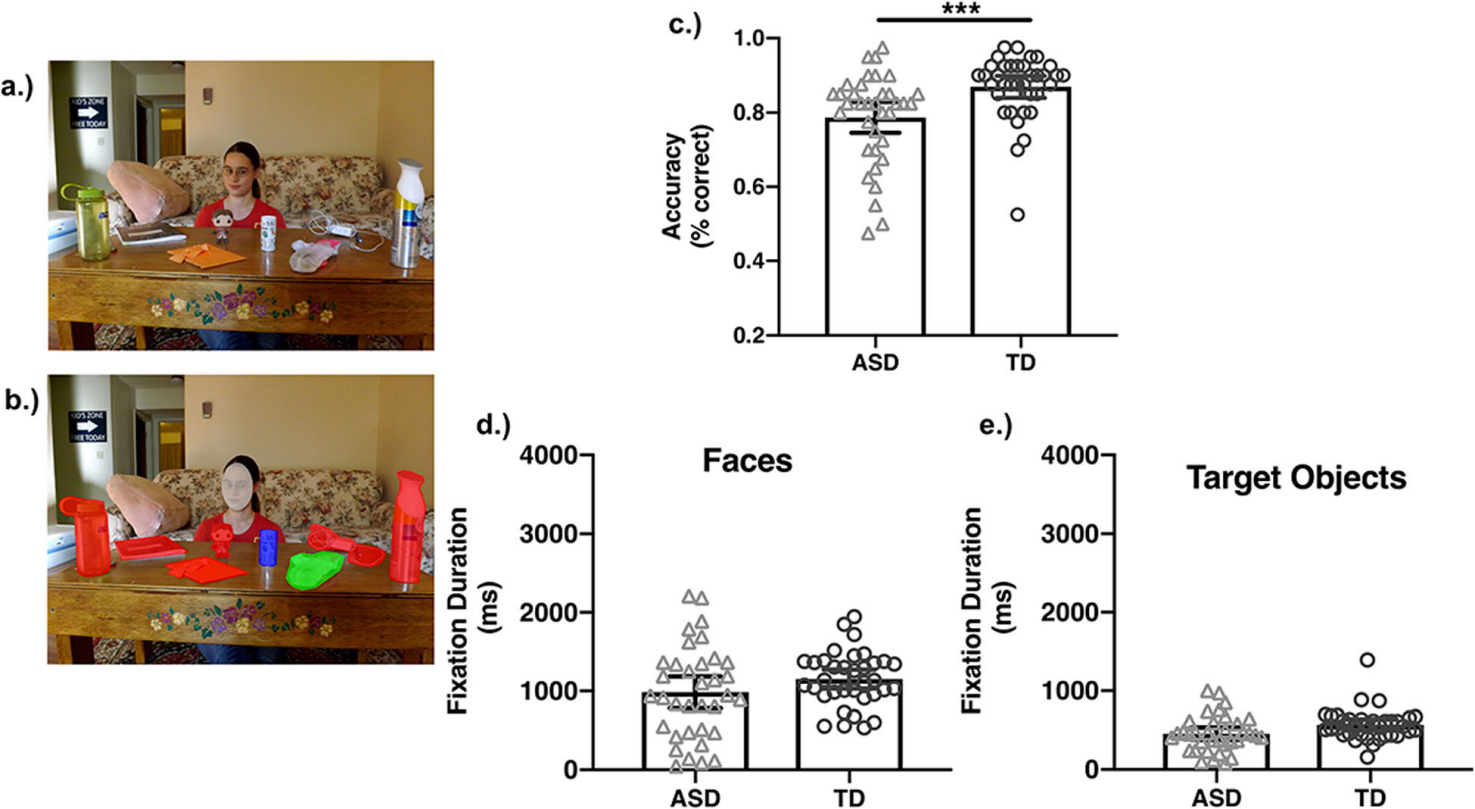
Gaze perception stimuli and results. Example image used in Gaze Perception Task (**a**). The areas of interest for the fixation analyses are illustrated in **b**. The target object is in green, the plausible non-target object is in blue, and the implausible non-target objects are in red. The face is in grey. Task performance (% correct) is plotted as a function of group (**c**). Fixation durations to faces (**d**) and target objects (**e**) are also plotted separated by group. Error bars reflect 95% confidence intervals. All analyses used mixed-effects modeling of trial-level data; however, for visualization these plots reflect the mean-level data. ***p < .001

Figure 3c shows the mean accuracy to identify the gazed-at object in the task as a function of group. As in the Gaze Following task, adolescents with ASD showed a relative impairment in the ability to identify target objects. There was a significant main effect of group (see Table 3). The variance of task performance was comparable across groups, *F*(68,1) = 2.21, *p* = .14.

#### Eye tracking data quality

The percentage of captured eye gaze samples was reduced in participants with ASD (*M* = 91%, SD = 8%) compared to TD (*M* = 95%, SD = 5%) participants (*b* = − 0.04, se = 0.02*,* 95% CI [− .08, − 0.01], *p* = .02).

#### Visual attention

Figure 3d, e shows the mean fixation duration to faces and target objects as a function of group.

#### Task engagement

Adolescents with ASD spent less time fixating the stimuli (*M* = 2594 ms, SD = 779 ms) compared to TD (*M* = 2878 ms, SD = 349 ms) adolescents (*b* = − 0.28, se = 0.14, 95% CI [− 0.57, 0], *p* = .05). The variance of these total fixation times was also different across groups, *F*(68,1) = 8.26, *p* = .005 (see Supplementary Figure 1). As a result, we included total fixation time for each stimulus as a covariate to control for this group difference in task engagement.

#### Faces

In contrast to predictions, adolescents with ASD and TD adolescents exhibited comparable fixation durations to faces (see Fig 3d). There was no main effect of group (see Table 3). This indicates that on average, adolescents with ASD and TD adolescents share similar amounts of social visual attention to faces. However, we did observe a significant difference in the variance of fixation durations to faces, *F*(68,1) = 8.63, *p* = .04. There was larger variance among the adolescents with ASD, indicating larger individual differences in looking times to faces (see Supplementary Figure 1).

#### Target objects

Similarly, adolescents with ASD and TD adolescents showed similar fixation durations to target objects (see Fig. 3e). There was no main effect of group (see Table 3), indicating similar allocation of amount of attention to target objects across groups. There were also no group differences in the variance of these fixation durations to target objects, *F*(68,1) = 1.00, *p* = .31 (see Supplementary Figure 1).

#### Relating eye tracking to behavioral data

**Fig. 4:**
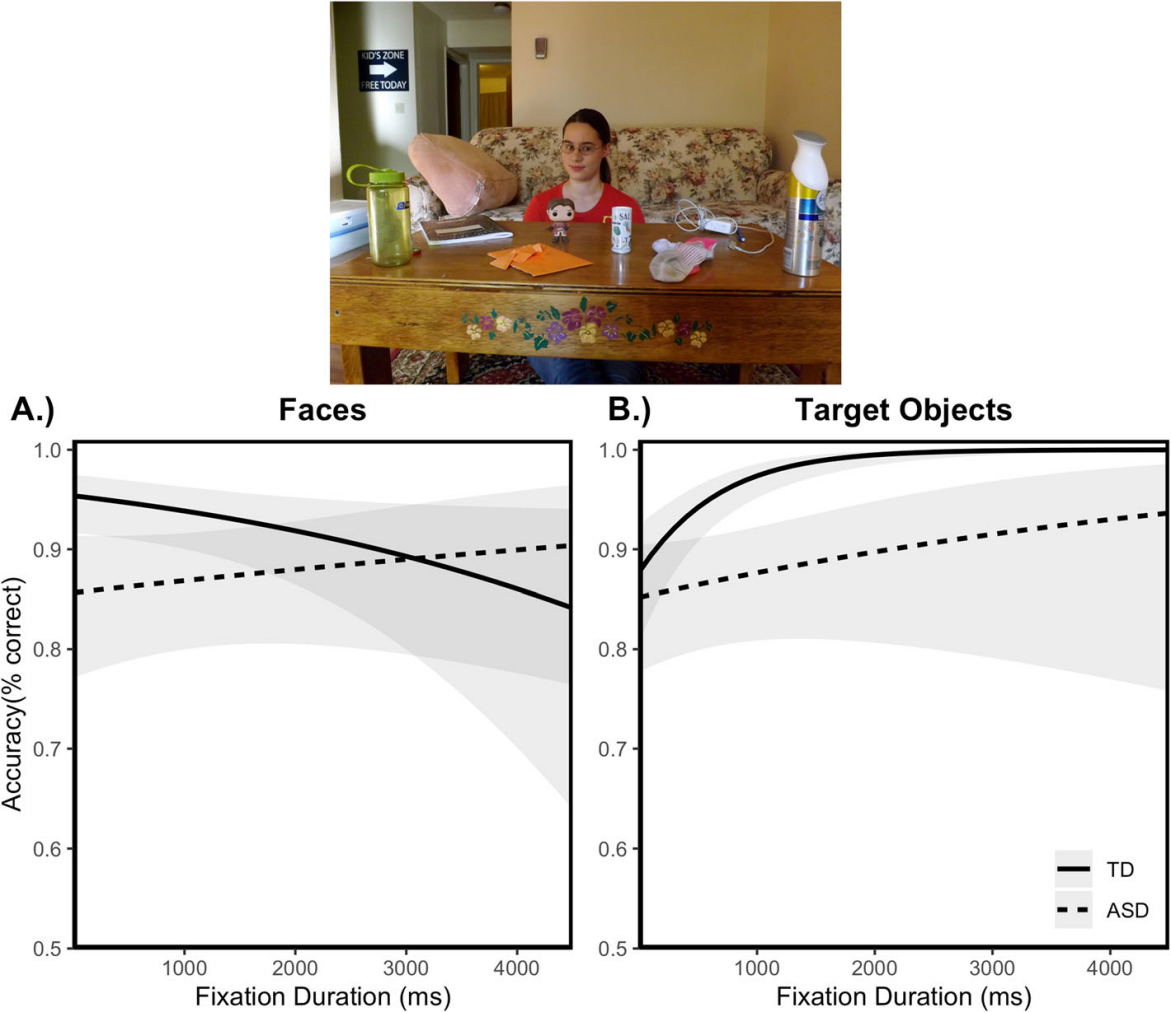
Association between visual attention and behavior during gaze perception. Probability of identifying correct target object as a function of fixation duration to *face*, plotted as a function of group (**a**). Shorter fixation durations to faces tended to be associated with better performance only in the typically developing adolescents. Probability of identifying correct target object as a function of fixation duration to *target object*, plotted as a function of group (**b**). Longer fixation duration to target objects was associated with improved task performance, but only for the TD adolescents. Shaded region reflects 95% confidence intervals. All plots reflect model-predicted relationships based on the mixed-effect models

In contrast to the Gaze Following task, total fixation duration to the stimuli did not predict task performance; there was no main effect of fixation duration. Importantly, there was also no fixation duration × group interaction, indicating that this association was not differentially present in either group (see Table 3). Figure 4 shows the association between looking time to face (a) and target object (b) AOIs and performance accuracy as a function of group.

#### Faces

There was no main effect of fixation duration on task performance. However, there was a fixation duration × group interaction (see Table 3). Fixation duration to faces was unrelated to task performance among the adolescents with ASD (Fig. 4a). However, among the TD adolescents, there was a trend for a negative association between looking time to the face and accuracy (see Fig. 4a). TD adolescents who looked at faces for the least amount of time tended to be more successful at identifying the target object in the 4AFC task.

#### Target objects

There was a main effect, which was qualified by a fixation duration × group interaction (see Table 3). There was no main effect among the adolescents with ASD (Fig. 4a). However, there was a positive main effect of fixation duration to the target object in the TD adolescents (see Fig. 4b), indicating that longer looking times to the target object were associated with correct responses in the 4AFC task.

#### Relating clinical assessments to eye-tracking and behavior data

Similar to the Gaze Following Task, there was a trend showing that ADOS-2 Total CSS scores negatively predicted fixation duration to target objects (*b* = − 0.04, se = 0.02, 95% CI [− 0.09, − 0.00], *p* = .06). Adolescents with ASD that had more severe autism looked less at the target objects. No other measures of social skills, autism symptoms, or problematic behaviors predicted task performance or fixation duration to the stimuli, faces, or target objects (see Supplemental Table 2).

## Discussion

We evaluated whether reduced visual attention to faces explains the difficulty processing eye gaze information in ASD. Specifically, we tested the prediction that limited looking time to faces predicts poor performance in the ability to identify the referential intent of eye gaze cues. This hypothesis builds on the clinical observation of reduced eye contact, particularly in social interactions, which could lead to lost opportunities to learn about eye gaze cues.

We employed eye-tracking technology to measure visual attention to faces and a 4AFC paradigm to measure behavioral responses in two tasks. We compared performance of adolescents with ASD and TD adolescents. In the Gaze Following task, we assessed group differences in the ability to follow and interpret the communicative intent of online eye gaze shifts. In the Gaze Perception task, we evaluated group differences in the ability to interpret eye gaze cues from static images involving a single actor among many objects. In both tasks, we also measured visual attention to the intentional locus of the gaze, the target object, and the association between visual attention and accuracy in task performance. In so doing, we assessed whether limited visual attention to faces and/or target objects is an underlying mechanism of impaired eye gaze processing.

### Following eye gaze for referential understanding

First, we evaluated whether adolescents with ASD exhibit a difficulty following online shifts in eye gaze to understand what another person intends to look at and communicate information about. The stimulus videos were designed much like those used in infant studies to assess joint attention skills [20, 21]. As predicted, adolescents with ASD exhibited a relative impairment in the ability to identify the target object, which is consistent with previous work describing children with ASD viewing similar video stimuli [21]. This impairment was equivalent to *one full standard deviation below* the TD group mean and reflects the difficulty adolescents with ASD have in following shifts in gaze for the purpose of referential understanding. In other words, the impaired performance reflects a difficulty interpreting the social communicative intent of the shift in gaze. This interpretation is supported by our finding that adolescents with ASD who were rated to have better social skills by their parents also performed better in this task.

Next, we evaluated the possibility that limited visual attention to faces influenced this impaired ability to understand eye gaze shifts. In contrast to predictions, adolescents with ASD and TD adolescents exhibited comparable fixation durations to faces while watching these videos of online gaze shifts. This indicates that at the group level adolescents with ASD allocate visual attention to faces and target objects similarly to TD adolescents. Importantly, we observed this similarity in social visual attention to faces after controlling for group differences in general task engagement. However, we did observe that the variability in fixation durations to faces was larger among the adolescents with ASD. This finding suggests that there are greater individual differences among adolescents with ASD in their social visual attention to faces during this task than there are among TD adolescents. Interestingly, these individual differences in social visual attention to faces are not related to autism severity, autism symptoms, or social behavior among the adolescents with ASD. These findings converge with those from infant [20], toddler [29], and child [21] studies reporting no differences in visual attention to faces at the group level. We also found that fixation durations to target objects were comparable in the ASD and TD groups (as was the variance among these durations) in the Gaze Following Task. In contrast to social visual attention to faces, the severity of autism as measured by ADOS-2 Total CSS scores predicted visual attention to the target objects. All together, these findings indicate that social visual attention to faces is not characteristically atypical in ASD and that gaze following behavior is intact in ASD.

One way to interpret our findings is to consider the component processes involved in gaze following. Following the gaze to the target object is a prerequisite for being able to interpret the referential intent of the gaze shift. However, while gaze following alone does not require an ability to represent another’s mental state (see [30, 31]), the ability to interpret the referential intent of a gaze shift does require mentalizing. Therefore, our findings may reflect that adolescents with ASD are capable of following gaze, but have difficulty linking gaze shifts with mental state information. Our findings relating looking times to behavioral performance in this task support this interpretation.

First, we assessed the association between target engagement and the ability to identify the target object in each group. Indeed, longer looking times predicted more accurate target identification in both groups and *to the same extent*. This is an important finding because it shows that task engagement is associated with better task performance in both groups equally. Next, we determined if visual attention to faces is associated with greater accuracy of target identification. The prediction was that short looking times would be associated with worse performance in the task. There was no such association between task performance and visual attention to faces in either group. This finding indicates that the duration of visual attention to faces is not related to the quality of information processing about gaze and challenges the notion that limited visual attention to faces underlies the difficulty processing eye gaze cues in autism.

We also measured the association between visual attention to target objects and the ability to identify these objects in the 4AFC task. In contrast to social visual attention to faces, longer looking times to target objects were associated with *enhanced* performance in the 4AFC task in both groups. In other words, limited visual attention to the target object, *not* the face, is a more plausible mechanism for explaining the difficulty understanding eye gaze cues in ASD. This interpretation is supported by our finding that the severity of autism is related to the duration of fixations to the target object. Together, these findings are consistent with the notion that the difficulty processing shifts in gaze in ASD is related to understanding the referential intent of the gaze shift. Understanding the social communicative nature of gaze shifts requires linking eye movements and mental state information about objects in the real world. Our findings suggest that adolescents with ASD had a much more difficult time making this connection.

### Perceiving eye gaze cues for referential understanding

We also evaluated whether adolescents with ASD exhibit an impairment in the ability to interpret eye gaze cues from photographs of complex scenes. Consistent with our findings from the Gaze Following task, adolescents with ASD evinced nearly a one standard deviation deficit in accuracy in the ability to identify the target object compared to the TD adolescents. This finding is consistent with that reported by Riby et al. [9] whose study and stimuli inspired the development of our task.

There were no group differences in looking time to either faces or target objects in the Gaze Perception task. This indicates that the allocation of visual attention during this task was also similar for adolescents with ASD and TD adolescents. These findings are inconsistent with those reported by Riby et al. [9]. Although we did find that adolescents with ASD looked at the stimuli less overall compared to TD adolescents, when we controlled for these task engagement differences, we discovered that the adolescents with ASD and TD adolescents were comparable in the way they allocated visual attention to these stimuli.

Recall that our goal was to test the assumption that reduced visual attention to faces contributes to the difficulty processing eye gaze cues. In contrast to predictions, the duration of looking time to faces did not predict the ability to pick out the correct label for the gazed-at object for adolescents with ASD. However, there was a trend for a *negative* association between looking times to faces and performance in the 4AFC task among TD adolescents. Specifically, individuals with the *shortest* looking times to faces tended to be the most successful at identifying the target object in the task.

This is especially interesting when considering the relation between looking times to the gazed-at object and accuracy in the 4AFC task. Again, there was a different pattern of association in each group. Among the adolescents with ASD, there was no association between looking times to target objects and the ability to identify them in the 4AFC task. In contrast, there was a positive association between looking times to the target object and the ability to identify them in the 4AFC task among the TD adolescents.

In sum, no variations in looking time to either faces or objects predicted performance for the adolescents with ASD. However, short looking times to faces and long looking times to target objects predict the best accuracy to interpret eye gaze cues among the TD adolescents. One interpretation of these findings is that long looking times to the face for TD adolescents may reflect difficulty computing the trajectory of eye gaze to the target object, whereas long looking times to the target object reflect an understanding of the trajectory and the referential intent of the gaze. The lack of an association between looking times and performance in this task for the adolescents with ASD indicates the difficulty in processing gaze information, especially for referential understanding.

## Limitations

The adolescents with ASD in this study were recruited into a larger intervention study that had inclusion criteria requiring that participants could be compliant with these testing procedures [16]. As a result, additional work is necessary to determine whether these novel results generalize to characterize individuals across the full spectrum of autism, including those with more prominent autism symptomology and lower IQ scores.

Also, the intervention study dictated the number of participants with ASD. It is important to note that while this study employed a sample size (*N* = 70) that is larger than the vast majority of existing studies of social visual attention in ASD as measured using eye-tracking, we acknowledge that expectations are changing regarding sample sizes in studies of ASD. This speaks to concerns about whether our experimental design was underpowered to detect group differences in social visual attention to faces. Critically, improving sensitivity to detect an effect is accomplished in two ways: by (1) increasing the sample size and/or (2) increasing sensitivity of the measures (e.g., decreasing error/measurement noise). Recall that we employed a within-subjects design and evaluated social visual attention to faces in two separate tasks (Gaze Following, Gaze Perception) using state-of-the-art eye-tracking technology and analyses. This design provided internal replication of our findings that social visual attention to faces in ASD is not characteristically deficient or explains difficulty in eye gaze processing. Therefore, despite the relatively smaller sample size by newer field standards, we have conducted the most methodologically rigorous test of the role of social visual attention to faces in deficient eye gaze processing in ASD. Moving forward, we encourage researchers to use a similar methodological approach with a larger sample size in the future. All of the stimuli are publicly available for research purposes [19].

Recent evidence indicates that among TD adults who vary in the broad autism phenotype, there are relative impairments in eye gaze processing but only among males [32]. This work led to the hypothesis that abnormal eye gaze processing may not be a diagnostic feature of autism in females. Unfortunately, we could not evaluate sex differences in visual attention, eye gaze processing, or referential understanding of gaze cues in this sample because we did not have the power to do so.

Finally, given the subtle differences in the patterns of results across the two tasks, it will be essential going forward to systematically evaluate the component processes (e.g., compute gaze trajectory, follow gaze, interpret communicative intent of gaze shift) of each task to determine which are particularly difficult for individuals with ASD. Based on our findings, we suggest that computing gaze trajectory and online gaze following appear to be fairly intact, while understanding the referential intent of eye gaze is impaired. Going forward, it will be important that new paradigms are developed to parametrically manipulate these component processes of eye gaze tasks to confirm this interpretation.

## Conclusion

This study tested the assumption that decreased visual attention to faces is an underlying mechanism of impaired eye gaze processing in ASD. We found no evidence to support this idea. Instead, visual attention to the locus of the gaze shifts, the target object, was more predictive of referential understanding. These findings suggest that the fundamental difficulty in autism is the referential understanding of gaze cues, which may not be extracted from visual cues and the distribution of visual attention alone. It requires linking information about mentalizing together with gaze information. The implication is that the common goal of many interventions, to increase looking time to faces, may not be helpful for improving understanding about eye gaze information. Instead, we recommend the emphasis be on helping individuals with ASD develop more referential understanding of gaze cues by identifying the locus of gaze shifts and linking that information to referential intent.

## Supplementary information

**Additional file 1:Supplementary** **Text 1:** Eye-tracking Protocol Orientation. **Supplementary Table 1**: Eye-tracking protocol and timeline. **Supplementary Table 2**: Parameter estimates for models assessing the influence of clinical measures (ADOS-2 Total CSS, SRS-2 Total, SSIS–Problem Behavior, SSIS–Social Skills) on task performance and visual attention to stimuli, faces, and target objects in the ASD group. **Supplementary Table 3**: Correlation Matrix for clinical measures in the ASD group.

**Additional file 2.Supplementary Figure 1**:Distribution of visual attention to stimulus items, faces, and target objects in ASD and TD groups.

## Data Availability

The datasets used and/or analyzed during the current study are available from the corresponding author on reasonable request.

## Abbreviations

ASD: Autism spectrum disorders
TD: Typically developing
4AFC: 4-alternative forced choice
ADOS-2: Autism Diagnostic Observation Schedule (2nd Ed.)
SRS-2: Social Responsiveness Scale (2nd Ed.)
CSS: Calibrated Severity Score
VIQ: Verbal IQ
FSIQ: Full scale IQ
NIQ: Non-verbal IQ
