# Supplementary material for: Does decreased visual attention to faces underlie difficulties interpreting eye gaze cues in autism?
Source: Mol Autism. 2020 Jul 21;11:60. doi: 10.1186/s13229-020-00361-2 (PMC7374971; doi:10.1186/s13229-020-00361-2)
Supplement: Supplementary file 1 — Additional file 1:Supplementary Text 1: Eye-tracking Protocol Orientation. Supplementary Table 1: Eye-tracking protocol and timeline. Supplementary Table 2: Parameter estimates for models assessing the influence of clinical measures (ADOS-2 Total CSS, SRS-2 Total, SSIS–Problem Behavior, SSIS–Social Skills) on task performance and visual attention to stimuli, faces, and target objects in the ASD group. Supplementary Table 3: Correlation Matrix for clinical measures in the ASD group. [file 13229_2020_361_MOESM1_ESM.pdf]

Supplementary Materials

Eye-tracking Protocol Orientation

The goals of this orientation procedure were to familiarize participants with the experimenter and room in which the eye gaze tasks were administered, provide participants with clear expectations about the sequence and duration of the eye gaze tasks, and help them understand how the eye tracking unit works to optimize data collection procedures (e.g., keeping the head fairly still). The orientation began with the researcher providing the adolescent participant with a verbal and visual description of the timeline of tasks and breaks (see Table 2). For each task, the researcher provided an age-appropriate description of the event (e.g., “First, you will watch a short series of movie clips and then we will take a short break. We will get up and leave the room to take a walk during the break to rest your eyes.”). Next, the researcher introduced participants to the eye-tracker, by naming it “Tobii the Robot” and explained that Tobii keeps track of eye movements. Participants were told that they need to be still so that Tobii can do its job. During this part of the orientation procedure, the eye-tracker was turned on so that the participant’s eyes could be tracked live to provide feedback about how their eye movements were being measured. Participants were invited to sit in a high-back office chair that was positioned in front of the eye tracking computer by matching the chair arms to specific locations on the computer table. Participants were told that they needed sit comfortably in the chair and be still during the tasks so that Tobii can do its job. Finally, the experimenter explained the calibration procedure, instructing participants to follow the small dot across 9 locations on the screen with their eyes while Tobii measured their eye movements.

Supplementary Table 1. Eye-tracking protocol and timeline

Order of Procedures	Calibration #	Time (minutes)
Eye-tracking Orientation		10
Social visual attention	Calibration 1	10
2-minute break		2
Eye Gaze Perception task: Part 1	Calibration 2	10; 20 trials
2-minute break		2
Eye Gaze Perception task: Part 2	Calibration 3	10; 20 trials
2-minute break		2
Eye Gaze Following task: Part 1	Calibration 4	10; 13 trials
2-minute break		2
Eye Gaze Following task: Part 2	Calibration 5	10; 13 trials
2-minute break		2
Social visual Attention Task 2	Calibration 6	3
Debrief		

Supplementary Table 2. Parameter estimates for models assessing the influence of clinical measures (ADOS-2 Total CSS, SRS-2 Total, SSIS – Problem Behavior, SSIS – Social Skills) on task performance and visual attention to stimuli, faces, and target objects in the ASD group.

	b/OR	***SE***	***t***	**Lower CI**	**Upper CI**	***p***
**Gaze Following Task**						
Task performance						
ADOS-2 Total CSS	0.82	0.09	-1.90	0.66	1.01	.06
SRS-2 Total	0.98	0.02	-0.82	0.95	1.02	.41
SSIS – Social Skills	**1.03**	**0.01**	**2.78**	**1.01**	**1.06**	**.005**
SSIS – Problem Behavior	0.99	0.01	-0.15	.97	1.03	.89
Visual Attention to Stimuli						
ADOS-2 Total CSS	-0.37	0.19	-2.01	-0.73	-0.01	.053
SRS-2 Total	-0.02	0.04	-0.56	-0.09	0.05	.58
SSIS – Social Skills	0.03	0.02	1.10	-0.02	0.07	.28
SSIS – Problem Behavior	-0.01	0.03	-0.54	-0.06	0.04	.59
Visual Attention to Faces						
ADOS-2 Total CSS	-0.24	0.17	-1.45	-0.57	0.09	.16
SRS-2 Total	-0.03	0.03	-0.88	-0.88	0.03	.38
SSIS – Social Skills	0.03	0.02	1.36	-0.01	0.07	.19
SSIS – Problem Behavior	-0.02	0.02	-1.09	-0.07	0.02	.29
Visual Attention to Target Object						
ADOS-2 Total CSS	**-0.10**	**0.04**	**-2.53**	**-0.18**	**-0.02**	**.02**
SRS-2 Total	0.01	0.01	1.14	-0.01	0.02	.26
SSIS – Social Skills	0.00	0.01	0.78	-0.01	0.01	.44
SSIS – Problem Behavior	0.01	0.01	1.89	-0.0003	0.02	.07
**Gaze Perception Task**						
Task Performance						
ADOS-2 Total CSS	1.15	0.10	1.62	0.97	1.35	.11
SRS-2 Total	1.02	0.02	1.22	0.99	1.05	.22
SSIS – Social Skills	0.98	0.01	-1.59	0.97	1.00	.11

SSIS – Problem Behavior	1.01	0.01	1.07	0.99	1.03	.28
Visual Attention to Stimuli						
ADOS-2 Total CSS	-0.13	0.07	-1.82	-0.28	0.01	.08
SRS-2 Total	-0.01	0.01	-0.1	-0.04	0.01	.33
SSIS – Social Skills	0.01	0.01	1.13	-0.01	0.03	.27
SSIS – Problem Behavior	-0.01	0.01	-0.83	-0.03	0.01	.42
Visual Attention to Faces						
ADOS-2 Total CSS	-0.08	0.06	-1.49	-0.20	0.03	.15
SRS-2 Total	-0.00	0.02	-0.30	-0.02	0.02	.77
SSIS – Social Skills	0.00	0.01	0.40	-0.01	0.02	.69
SSIS – Problem Behavior	-0.00	0.01	-0.43	-0.02	0.01	.67
Visual Attention to Target Object						
ADOS-2 Total CSS	-0.04	0.02	-1.99	-0.09	-0.00	.06
SRS-2 Total	0.00	0.00	0.42	-0.01	0.01	.68
SSIS – Social Skills	0.00	0.00	1.21	-0.00	0.01	.23
SSIS – Problem Behavior	0.00	0.00	0.81	-0.003	0.01	.42

Note. ADOS-2 = Autism Diagnostic Observation Schedule (2nd Edition); SRS-2 = Social Responsiveness Scale; SSIS = Social Skills Improvement System; CSS = Calibrated Severity Score

Supplementary Table 3. Correlation Matrix for clinical measures in the ASD group

	ADOS-2 Total CSS	ADOS-2 SA	ADOS-2 RRB	SSIS Problem Behavior	SSIS Social Skills	SRS-2 Total
ADOS-2 Total CSS	1	0.83	0.70	0.08	-0.13	0.08
ADOS-2 SA		1	0.31	0.06	-0.16	0.07
ADOS-2 RRB			1	0.01	0	-0.07
SSIS Problem Behavior				1	-0.44	0.76
SSIS Social Skills					1	-0.75
SRS-2 Total						1

Note. ADOS-2 = Autism Diagnostic Observation Schedule; SRS-2 = Social Responsiveness Scale; SSIS = Social Skills Improvement System, ASD = Autism Spectrum Disorder; SA = Social Affect; RRB = Restricted and Repetitive Behavior
