# Supplementary material for: Does decreased visual attention to faces underlie difficulties interpreting eye gaze cues in autism?
Source: Mol Autism. 2020 Jul 21;11:60. doi: 10.1186/s13229-020-00361-2 (PMC7374971; doi:10.1186/s13229-020-00361-2)
Supplement: Supplementary file 2 — Additional file 2.Supplementary Figure 1:Distribution of visual attention to stimulus items, faces, and target objects in ASD and TD groups. [file 13229_2020_361_MOESM2_ESM.pdf]

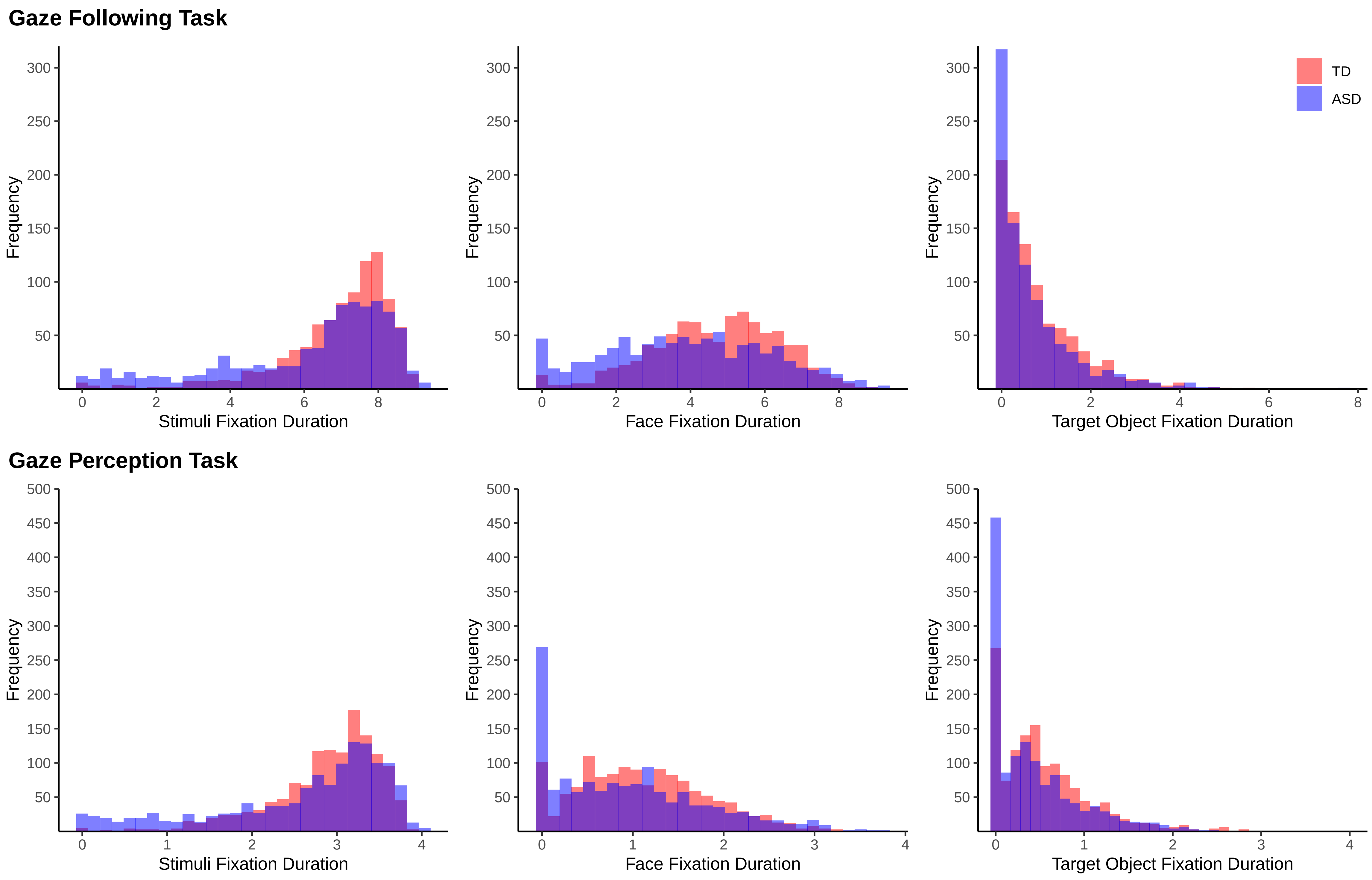

Supplementary Figure 1. Distribution of visual attention to stimulus items, faces, and target objects in ASD and TD groups. Fixation Duration is in seconds.
